# Homœopathy in Its Relation to the Diseases of Females

**Published:** 1887-03

**Authors:** 


Homœopathy; in its Relation to the Diseases of Females, or Gynæcology. By Thomas Skinner, M. D. London. The Homœopathic Publishing Co. 1886.

This is the third edition of that remarkable little book first issued by Dr. Skinner in 1875, on his joining the homœopathic school. As every one in the profession is familiar with it, it is unnecessary for us to discuss its merits. We therefore content ourselves with a simple announcement that this is the *third* edition. W. M. J.

